# Degree of handedness and priming: further evidence for a distinction between production and identification priming mechanisms

**DOI:** 10.3389/fpsyg.2015.00151

**Published:** 2015-02-19

**Authors:** Donna J. LaVoie, Brianna Olbinski, Shayna Palmer

**Affiliations:** Department of Psychology, College of Arts and Sciences, Saint Louis University, St. Louis, MO, USA

**Keywords:** priming, handedness, hemispheric communication, memory, task dissociations

## Abstract

The distinction between implicit and explicit forms of memory retrieval is long-standing, and important to the extent it reveals how different neural architecture supports different aspects of memory function. Similarly, distinctions have been made between kinds of repetition priming, a form of implicit memory retrieval. This study focuses on the production–identification (ID) priming distinction, which delineates priming tasks involving verification of stimulus features as compared to priming tasks that require use of a cue to guide response retrieval. Studies investigating this dissociation in dementia or similar patient populations indicate that these forms of priming may differ in their neural bases. The current study looks at degree of handedness as a way of investigating inferred neural architecture supporting these two forms of priming. A growing body of research indicates that degree of handedness (consistent, or CH, versus inconsistent, or ICH) is associated with greater interhemispheric interaction and functional access to right hemisphere processing in ICH, with superior performance seen in ICH on memory tasks reliant on this processing. Arguments about the theoretical mechanisms underlying ID and production forms of perceptual priming tasks suggest that performance on these tasks will differ as a function of degree of handedness. We tested this question in a group of CH and ICH young adults, who were asked to study lists of words prior to performing a production priming task (word stem completion, WSC), a perceptual word ID task, and a word stem cued recall task. While both handedness groups exhibited reliable priming across tasks, WSC priming was greater in ICH than CH participants, with ID priming not differing between groups. This dissociation supports the argument that production and ID forms of priming have different underlying neural bases.

## INTRODUCTION

The fact that memory expresses itself in diverse ways is well established. For example, memory can be expressed explicitly or implicitly. Explicit memory has been described as memory that includes conscious, deliberate retrieval of past experiences or information, and implicit memory has been described as an unintentional form of retrieval occurring without awareness. Explicit memory is measured through the use of recall or recognition tests, in which individuals are asked to deliberately retrieve information from a target experience or study episode. Implicit memory, on the other hand, is typically measured through repetition priming, where the contents of memory are revealed through facilitated performance (greater accuracy, faster responding) on tasks using previously encountered materials relative to new materials, without deliberate conscious recollection of a target episode or event. Dissociations between explicit and implicit memory performance are often observed, especially in amnesia (e.g., Shimamura, 1986) and aging (e.g., LaVoie and Light, 1994), where amnesics and older adults often demonstrate impaired performance on explicit memory tasks relative to normal controls and young adults, respectively, yet relatively spared performance on repetition priming tasks. Such dissociations are used to support the argument that different memory systems or underlying neural architecture support these different forms of memory.

Just as there are observed dissociations between tasks used to measure implicit and explicit memory, dissociations also have been observed between different categories of tasks typically used to measure repetition priming. One observed dissociation is between perceptual and conceptual forms of priming (Roediger and McDermott, 1993). Perceptual priming reflects memory for stimulus form, and is hypothesized to engage perceptual processing mechanisms. As a result, this form of priming is sensitive to perceptual characteristics of stimuli across study-test conditions (e.g., there is reduced priming when the stimulus study and test modality differ). Examples of perceptual priming tasks include word identification (ID), picture or word naming, word stem and word fragment completion. Conceptual priming refers to memory for stimulus meaning, and is sensitive to manipulations that enhance semantic processing, e.g., conceptual elaboration of study words, but is rather impervious to perceptual characteristics of stimuli.

Another dissociation, and the focus of this study, is that between production versus ID priming (Gabrieli et al., 1994). ID priming tasks are typically those in which an individual must identify, classify, or verify an attribute of a stimulus from a guiding cue, with the response limited by the cue to a single item. For example, in word ID tasks individuals must identify a briefly flashed word. Priming is exhibited when previously studied words are more readily identified (i.e., more quickly or with greater accuracy) than new words. In this case, the guiding cue is the word itself. Production priming tasks are those in which an individual uses a cue to produce a response, but that response is not limited to a single specific item. Rather, a target item must be selected from amongst an array of plausible alternatives. For example, in word stem completion (WSC) tasks, individuals must add letters to the first three letters of a word cue to form a longer word. Priming is exhibited when the added letters form a previously studied word versus an alternative completion.

Dissociations between ID and production based priming tasks are hypothesized to be due to the differing demands on attentional resources that each of these tasks makes. Relative to ID tasks, production tasks involve high levels of response competition due to the cueing of more than one alternative, with resolution of this competition necessary to produce a response. Gabrieli et al. (1999) argue that attentional resources are essential at encoding for the target item to be produced instead of an alternative. In support of their argument, they report reduced priming on production tasks relative to ID tasks in individuals with Alzheimer’s disease, a disease in which attention deficits manifest early in its course, as well as in young adults experiencing divided attention during study. Others have reported similar production–ID dissociations (e.g., Fleischman et al., 2001; LaVoie and Faulkner, 2008), although dissociations are not always observed, even when stimuli are chosen so that response competition is directly manipulated, and demands on attention theoretically increased (e.g., Geraci and Hamilton, 2009; Prull, 2010). Nonetheless, a recent meta-analysis examining the relationship between divided attention and implicit memory performance across 21 studies supports the production–ID priming distinction, with divided attention effects being larger for production tasks than ID tasks (Spataro et al., 2011).

The reported role of attention in production based priming tasks is particularly important to the study described here, as arguments have been made (e.g., Banich and Belger, 1990) that as task complexity (and consequent demands on attentional resources) increases, interhemispheric interaction becomes important for task performance. Specifically, interhemispheric interaction is argued to be a general strategy employed by the brain to increase attentional resources when task demands are high (Passarotti et al., 2002). Support for this argument rests largely in the use of the Banich paradigm, in which participants are asked to gaze at a fixation point on a screen, with a target letter displayed at the bottom, to either the left or right side of the fixation point. Probe items are displayed at the top of the screen, also to the left and right of the fixation point. On all trials, participants are to decide if the target and probe items match. In within-hemisphere conditions, the probe and target letters appear on the same side of the screen, in their respective rows (e.g., both left of the fixation point). Matching decisions on these trials is assumed to occur without interhemispheric interaction as both the target and probe are encoded within the same hemisphere. In across-hemisphere conditions, target, and probe items appear on opposite sides of the fixation point in their respective rows (e.g., the target is left of the fixation point, but the probe is right of the fixation point). Match decisions on these trials require interhemispheric interaction as the probe and target items are encoded in different hemispheres. Critically, as task demands increase, performance improves on across-hemisphere trials, but does not on within-hemisphere trials (see Banich, 1998, for a review). This across-hemisphere advantage is argued to occur because processing resources are recruited from both hemispheres to meet difficult task demands, while such recruitment and coordination of processing across hemispheres on easier tasks produces a cost that negatively impacts performance on the easier trials. Interhemispheric interaction appears to be an important factor, then, in the performance of complex, attentionally demanding cognitive tasks, but is less important for the performance of simpler, less attentionally demanding cognitive tasks. This conclusion is particularly important to the study described here, as a growing body of research is pointing to a similarly important role of interhemispheric interaction in memory function.

The important role of interhemispheric interaction in memory function has come in recent years from the examination of degree of handedness differences in memory task performance. This research typically compares the performance of mixed (or inconsistent) handedness (ICH) to strong (or consistent) handedness (CH), hypothesizing that the larger corpus callosum size seen in ICH relative to CH individuals (Luders et al., 2010) produces decreased interhemispheric communication and poorer integration of information across hemispheres in CH individuals on tasks in which such interhemispheric communication is needed. As such, degree of handedness has been argued to be a marker for functional differences in memory performance on tasks hypothesized to be dependent upon interhemispheric interaction (Lyle et al., 2008), with ICH individuals hypothesized to perform better than CH individuals on such tasks. Indeed, across a variety of paradigms, ICH is associated with better performance on memory tasks, including superior source memory (Lyle et al., 2008), greater resistance to false recall (Christman et al., 2004), as well as greater incidental memory for deeply processed words (Christman and Butler, 2011). The majority of tasks testing this argument have been episodic memory tasks, but the finding that production priming tasks are sensitive to divided attention manipulations suggests that as a category of tasks, they too may be sensitive to degree of handedness, especially given the important role of interhemispheric interaction in attentionally demanding tasks. To the extent that production priming tasks are more demanding of attention than are ID tasks, and to the extent that interhemispheric interaction is necessary for performance of attentionally demanding tasks, then there should be differences between CH and ICH individuals on production priming tasks, but not ID priming tasks. While Propper et al. (2005) reported no handedness differences in fragment completion priming, a type of production priming task, it is difficult to make generalizations about priming performance and degree of handedness from this single study, especially when no other type of priming task was included. The purpose of the study described here, then, was to specifically examine production and ID priming performance in CH and ICH individuals, as we hypothesize that degree of handedness should reveal production–ID priming dissociations, given the demands production tasks make on attention and interhemispheric interaction. We expected CH individuals as compared to ICH individuals to demonstrate reduced priming on a WSC task, but equivalent priming between degree of handedness groups on a word ID task, a task not hypothesized to be as attentionally demanding and therefore not as reliant on interhemispheric interaction.

## MATERIALS AND METHODS

### PARTICIPANTS

Thirty-nine individuals (mean age 19.31 years, SD = 1.10), recruited from undergraduate psychology courses at Saint Louis University, participated. All received partial course credit for their participation. Twenty-four (61.5%) of the participants were male. Handedness was measured via the Edinburgh Handedness Inventory (EHI; Oldfield, 1971) where scores can range from –100 (exclusive left-handed) to +100 (exclusive right-handed), with a score ≥±80 used to distinguish consistent from inconsistent handedness (Prichard et al., 2013). Using these criteria for classification, and EHI scores in our total sample ranging from 30 to 100, 22 (56.4%) participants were categorized as consistent handed (CH; scores 80–100), and 17 participants were categorized as inconsistent handed (ICH; scores 30–75), to form two degree of handedness groups. Both groups scored similarly on tests of forward (M_CH_ = 6, M_ICH_ = 7) and backward (M_CH_ = 5, M_ICH_ = 5) digit span, as well as on the Nelson–Denny Vocabulary test (M_CH_ = 13/25, M_ICH_ = 14/25).

### STIMULI

Study stimuli consisted of 238 nouns, one to three syllables in length, selected from the MRC Psycholinguistic Database (Wilson, 1988). The average Kucera and Francis (1967) frequency rating of these words was 39 per million. Two hundred items were randomly chosen to appear as either study items (100 items) or filler items (100 items) in the study-test sessions, with their role as either study or filler item counterbalanced across conditions so that all words appeared in each of these roles across participants. An additional 100 items chosen using the same criteria were used as items for a perceptual threshold baseline task, and never appeared as study or filler items in the primary study-test tasks. E-Prime was used for the presentation of all stimuli in the computer-based tasks.

### PROCEDURE

The basic procedures of this experiment replicated those of LaVoie and Faulkner (2008). After being presented with the recruitment statement and answering any questions, participants were informed that they would be presented with a series of computer-based and paper-and-pencil tasks. Each participant was tested individually.

The initial task for all participants was the perceptual threshold baseline task, used to determine individual word ID thresholds. Using a staircase procedure, participants were asked to identify individual words presented on the computer screen at rates varying from 13 to 130 ms. Each presentation increased or decreased in 13 ms steps (depending on whether the current staircase was ascending or descending) in order to determine the presentation rate at which each participant could correctly identify approximately 50% of the items. Each trial began with an on-screen focal cue (++++++) lasting for 500 ms followed by a 100 ms blank screen and then a word (which stayed on screen for 13–130 ms). Each word was followed by a pattern mask (@@@@@@) lasting for 60 ms, followed by a string of question marks as a prompt for the participant to verbally identify the briefly presented word. Participants were encouraged to guess if they were uncertain. Ten staircase trials were included in this task in order to determine the display rate threshold for each individual participant, which was then utilized in the later word ID task. The mean display rate determined for each group using this procedure was identical at 26 ms (the range across individuals was 13–39 ms).

Two study-test sessions followed the perceptual threshold task. Each session started with the presentation of a study list, followed by a 10-min delay in which participants completed math problems to prevent study item rehearsal, a repetition priming task (either WSC or word ID; the order of which was counterbalanced across conditions), then a cued recall test. For the study lists, items were individually presented on-screen for 2000 ms with a 500 ms blank screen between items. In order to ensure proper encoding, participants were asked to recite each word aloud as it was presented. Participants then completed math problems for 10 min prior to performing the priming task, either the WSC task or word ID task. The paper-and-pencil WSC task consisted of 50 three-letter word stems (25 studied items, 25 unstudied fillers, randomly ordered). All stems could be completed to form more than one alternative. Participants were asked to add one or more letters in order to form the first word that came to mind, with no time constraint to complete the task (participants generally completed the task in under 5 min). For the computer-based ID task participants were presented a different set of 25 studied items and 25 unstudied fillers in random order, with each item presented at the display rate determined by the perceptual threshold baseline task at the start of the experiment. Participants were encouraged to verbally guess the word if they were unable to confidently identify it. The experimenter noted each response and initiated each individual trial.

The second study-test session immediately followed the first study-test session. Identical procedures were used with the only difference being the use of the priming task that was not included in the first session (to ensure counterbalancing). After completion of both study-test sessions, participants received a cued recall test consisting of 100 items (50 studied items not used on either of the priming tasks, 50 unstudied fillers). The first three letters of each item were given as cues and participants were instructed to specifically think back to items they had studied earlier in the experiment to complete the stems. Finally, participants received digit span measures (forward and backward), the EHI, a demographics questionnaire, and the Nelson–Denny vocabulary test. At the conclusion of all testing, participants were asked whether during performance of the priming tasks (i.e., the WSC and word ID tasks) they noticed that some of the words had been presented earlier (i.e., during the encoding/study phase). For those participants that responded affirmatively (*N* = 3), a follow up question was posed asking if they intentionally used those words to complete the stems during that priming task. No participant responded affirmatively to this question.

## RESULTS

All test score data (WSC, ID, and stem cued recall) were analyzed in separate independent group *t*-tests comparing CH to ICH participants. For the repetition priming tasks, the scores used for analysis consisted of priming scores: (1) for the WSC task, priming scores were calculated as studied items completed as targets minus filler items completed as targets; (2) for the ID task, priming scores were calculated as studied items correctly identified minus filler items correctly identified. For the stem cued recall task, the scores used for analysis were the proportion of studied word stems correctly completed as studied items. Mean proportion correct and priming scores for each task and group are displayed in Table 1. As predicted, WSC (production) priming was reliably greater in the ICH group than the CH group, *t*(37) = 2.156, *p* = 0.038 (effect size Cohen’s *d* = 0.697), while word ID priming did not differ between groups, *t*(37) < 1.0. We note that the magnitude of ID priming is relatively low in both groups [although in both groups the magnitude of priming is greater than 0, CH *t*(21) = 3.59, *p* = 0.002, ICH *t*(17) = 3.92, *p* = 0.001] with baseline ID rates being unexpectedly well above 50%, and indeed at ceiling levels of performance. If we remove from analysis those individuals who identified 100% of the ID filler items (10 CH individuals, six ICH individuals), a different pattern emerges, with CH (*n* = 12) individuals demonstrating greater ID priming (mean priming = 0.09, reliably greater than 0, *p* = 0.000) than ICH (*n* = 11) individuals (mean priming = 0.05, reliably greater than 0, *p* = 0.000), *t*(21) = 2.04, *p* = 0.054. (It should be noted that the between group difference on WSC priming performance remains, although is somewhat reduced; mean WSC priming CH = 0.10, mean WSC priming ICH = 0.15, *p* = 0.051). WS cued recall also did not differ between groups, *t* < 1.0 for both the full and reduced sample analyses.

**TABLE 1:** **Mean proportion correct and mean priming effects for each task by handedness (SD's in parentheses)**.

Task		CH	ICH
WSC	Studied target	0.32 (0.10)	0.42 (0.11)
	Filler	0.24 (0.08)	0.27 (0.08)
	Difference (priming)	0.08 (0.10)	0.15 (0.10)
ID	Studied target	0.99 (0.02)	0.99 (0.05)
	Filler	0.94 (0.07)	0.95 (0.03)
	Difference (priming)	0.05 (0.06)	0.04 (0.04)
	Cued recall	0.37 (0.07)	0.39 (0.09)

## DISCUSSION

As a reminder, the primary purpose of this study was to examine potential production–ID priming task dissociations between CH and ICH individuals. Our findings indicate such dissociation exists. In our complete full sample analyses, these groups show a single dissociation, with greater production priming shown in ICH relative to CH individuals, as we predicted, but equivalent levels of ID priming. In our smaller sample analyses, a double dissociation emerges, with ICH individuals continuing to demonstrate larger production priming effects than CH individuals, but CH individuals showing larger ID priming effects than ICH individuals. While preliminary, the overall pattern of findings we report extend both our understanding of production and ID priming, as well as our understanding of the role of degree of handedness in memory.

The extant findings showing systematic variations in explicit memory performance as a result of degree of handedness point to the role of interhemispheric communication as an important factor supporting memory function. To the extent that memory task performance is reliant on integration and comparison of hemisphere-specific information, then performance will be better when such integration occurs (Propper et al., 2005). Our findings suggest that this integration is important for production priming as well, and is consistent with reports in the literature examining the role of hemisphere-specific information on priming performance in patients with complete callosotomies (e.g., Cronin-Golomb et al., 1996; Kroll et al., 2003). In these studies, WSC priming appears dependent upon access to right hemisphere (RH) information, especially when multiple alternatives exist to form a completion. Cronin-Golomb et al. (1996) argue that the information transferred across hemispheres is necessary for the elicitation of an appropriate response from an array of limited, possible responses, and that without such information transfer, WSC priming is impaired. This information transfer may be necessary for attentionally demanding tasks only, as priming that involves single solutions (as may be the case in ID priming tasks where cues direct individuals to a single response) appears to be less reliant on integration of information across the two hemispheres. Our findings are generally consistent with these claims from patient studies, and further support distinctions in production–ID priming. Future research specifically comparing single versus multiple solution production task performance as a function of degree of handedness would be helpful in elucidating this argument.

Unexpectedly, stem cued recall did not differ between groups. The simple explanation for this finding is that we lacked the power to detect such a difference given our sample size, but we’d like to speculate on some potential alternative theoretical explanations. First, while degree of handedness generally produces group differences in recall performance, it is not always found on tasks like ours. Christman and Butler (2011), for example, employed a levels of processing manipulation in an incidental learning paradigm, and found that recall of words processed at shallow levels did not differ between CH and ICH individuals. The generally low levels of processing performed on study items in our task may be a factor in explaining why we did not observe group differences in cued recall. Both ours and the Christman and Butler (2011) findings suggest that degree of handedness effects in memory function may only be present when information is processed or encoded at a meaningful level, but more research is needed specifically examining this hypothesis before generalizations can be made. As a second alternative explanation, consider work conducted by Marsolek et al. (1992), in which they investigated the hypothesis that there are distinct word form brain systems involved in the performance of priming tasks (defined as WSC tasks in their set of experiments) and cued recall (defined as WS cued recall). They find evidence that the RH controls a word form system that is responsible for storage of form-specific representations of words that support priming on WSC tasks, affording a RH advantage in WSC priming. Both hemispheres, though, maintain abstract word forms that support recognition and WS cued recall, and so the RH advantage observed on WSC priming tasks is no longer observed. These findings suggest that degree of handedness differences may not be present when WS cued recall tasks are employed, as performance on such tasks is not RH dependent, but can be supported by left hemisphere processing. Our findings support such an argument, but remain purely speculative. Nonetheless, it does suggest further exploration in future studies to better delineate the conditions and factors by which degree of handedness impacts memory.

### CONFLICT OF INTEREST STATEMENT

The authors declare that the research was conducted in the absence of any commercial or financial relationships that could be construed as a potential conflict of interest.
